# A Better Understanding of Bee Nutritional Ecology Is Needed to Optimize Conservation Strategies for Wild Bees—The Application of Ecological Stoichiometry

**DOI:** 10.3390/insects9030085

**Published:** 2018-07-18

**Authors:** Michał Filipiak

**Affiliations:** Institute of Environmental Sciences, Jagiellonian University, ul. Gronostajowa 7, 30-387 Kraków, Poland; michal0filipiak@gmail.com or michal.filipiak@uj.edu.pl

**Keywords:** bee, pollen, ecology, nutrition, conservation, pollinator, decline, health, stressor, constraint

## Abstract

The observed decline in wild bees may be connected to the decreasing diversity of flowering plants. Changes in floral composition shape nutrient availability in inhabited areas, and bee larvae need food rich in body-building nutrients to develop into adults. Adult food, mainly composed of energy-rich nectar, differs from larval food, mainly composed of pollen, and adult bees forage on different plant species for nectar and pollen. Defining bee-friendly plants based on the quantities of food produced, and on the visitation rates of adult pollinating insects leads to the planting of bee habitats with poor-quality food for larvae, which limits their growth and development, and negatively affects the population. Consequently, failing to understand the nutritional needs of wild bees may lead to unintended negative effects of conservation efforts. Ecological stoichiometry was developed to elucidate the nutritional constraints of organisms and their colonies, populations, and communities. Here, I discuss how applying ecological stoichiometry to the study of the nutritional ecology of wild bees would help fill the gaps in our understanding of bee biology. I present questions that should be answered in future studies to improve our knowledge of the nutritional ecology of wild bees, which could result in better conservation strategies.

## 1. Background

### 1.1. An Improved Understanding of Wild-Bee Nutritional Ecology Is Needed

Pollinators are the key component of global biodiversity shaping natural-plant communities and supporting human food production. It was calculated that 80% of wild-plant species and 75% of all crops depend on insect pollinators [1,2,3]. Accordingly, pollination is repeatedly identified as a particularly valuable ecosystem service (e.g., References [1,2,4,5]). Its economic value was estimated at approximately €150 billion (equivalent to almost US $200 billion), accounting for 9.5% of the economic value of annual global food production [6]. However, pollinating insects, especially bees, are faced with growing pressures. These pressures result in losses in their numbers and diversity, referred to as global pollinator declines [3,5,7].

Disagreements emerged as to the accuracy of assessments of the loss of pollinators [8]. Some studies, focusing on species used in agriculture, provided insufficient evidence for pollinator decline. Their conclusions may be questioned. However, looking broadly across the variety of pollinators reveals clear evidence of their decline at the local, regional, and global scales, particularly for wild bees [7,8]. In a review of the roles of resources and risks in regulating wild-bee populations, Roulston and Goodell [9] determined that, among the variety of direct and indirect factors limiting bee populations (e.g., nesting resources, parasites, tilling, predators, pesticides, land management, landscape context, and invasive species), the availability of food resources is the most important factor. Another review focused on how floral resources shape bee populations and pollination services, and concluded that a refined understanding of wild-bee nutritional ecology is needed for predicting and managing bee-mediated pollination services [10].

Studies on pollinator biology and ecology mainly focus on *Apis mellifera* L. [7,11,12,13]. Several comprehensive reviews of the nutritional physiology and ecology of this species were published (Reference [14] with literature cited there), and according to my knowledge, a similarly detailed level of information does not exist for any other single bee species (however, we do have general knowledge on the physiology, ecology, and nutrition of several bee groups, e.g., Meliponini stingless bees [15]). The total number of bee species worldwide is estimated as 20,000. These bees inhabit various environments, have diverse life histories and feeding strategies, and experience different limitations shaped by bee-plant interactions. Failing to understand the diversity of bee feeding strategies might have implications for the effects of bee conservation, resulting in unintended changes in ecosystems.

Not all plants produce pollen the nutritional quality of which satisfies the nutritional requirements of bees [16,17]. Nonetheless, in choosing bee-friendly plants for planting, we tend to ignore their quality as food for bees. In particular, we tend to ignore larval nutritional needs which differ from adult requirements. Bee-friendly plants are often defined based on the quantities of nectar and pollen produced, and on the visitation rates of adult pollinating insects. However, bees forage on different plant species for nectar and pollen [18,19,20], and the species composition of the pollen used as larval food may influence bee survival [21]. Adult food, which is rich in energy, has different characteristics from larval food, which is rich in body-building matter. Wild bees may have almost infinite access to energy-rich food that meets the nutritional needs of energetically limited adults, yet a large quantity of food for bee larvae cannot compensate for poor quality. In such a way, poor nutritional balance may limit the growth and development of individuals, thus negatively influencing their populations. Therefore, to balance the bee diet and ensure that bee populations can thrive, we should pay special attention to pollen quality.

**Figure 1:**
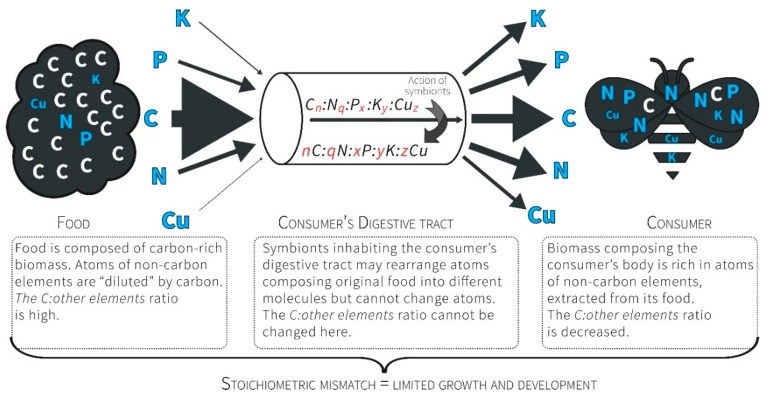
Consumers ingest a prepackaged ratio of atoms. For herbivores, the food contains more C relative to other atoms, so they must manage a diet that presents a stoichiometric mismatch through excess C, which is further exacerbated by the unbalanced relationships between non-C elements (due to the exceptional scarcity of some of them). Consumer graphic source: Vecteezy.com (https://www.vecteezy.com/vector-art/169213-flat-six-bees-vectors).

Consumers are limited in their juvenile life stage by the availability of molecules allowing for maximized growth. This limitation is imposed on the consumer’s growth and development, and, if not mitigated, results in underdeveloped adults, whose fitness is reduced. In extreme cases, such a limitation leads to the consumer’s death before it reaches the adult stage. Some of the molecules that do not compose the original food, but are needed by consumers, may be produced by gut symbionts. However, both consumers and their gut symbionts are limited if the diet is not stoichiometrically balanced. The reason is the law of conservation of mass, according to which the constituent atoms of nutrients cannot be created by organisms from the available biomass, and therefore, must be present in the diet in adequate proportions ([22,23], Figure 1).

The law of conservation of mass is crucial for ecological interactions occurring in the ecosystem, including plant–insect interactions [16], and bees and their host plants must have evolved within the frame of this law. The study of the balance of energy and matter in ecological interactions that capitalizes on consumers and their food to be composed of identical building blocks—atoms of chemical elements—is called ecological stoichiometry [22,23].

### 1.2. The Framework of Ecological Stoichiometry May Be Applied for A Better Understanding of Bee Nutritional Ecology

**Figure 2:**
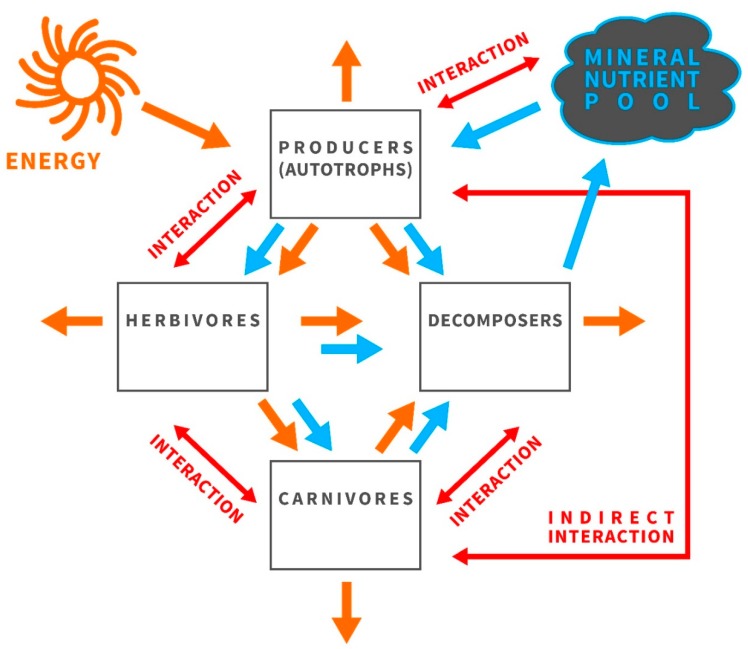
Organisms in an ecosystem interact with each other and with the abiotic world to grow, develop, produce progeny, and maximize fitness. Energy, shown in orange, is flowing through the ecosystem while the mineral pool (in blue) is cycling, and consumers are drivers of this cycle. Only a given pool of nutritional supply is available, and interactions between components of this food web (in red) are shaped by food quality, which is crucial for consumer growth and population dynamics. The study of the balance of energy and matter in ecological interactions is called ecological stoichiometry. This research framework capitalizes on the fact that organisms are composed of identical building blocks—atoms of chemical elements—even though they build remarkably diverse organic molecules. Within this context, it is possible to focus on a single interaction within this web.

The framework of ecological stoichiometry was developed to better understand the nutritional constraints on growing and developing organisms, and how these constraints shape ecological interactions [22]. In contrast to the majority of ecological analyses, which are based on single dimensions (energy, biomass, nitrogen, or carbon), ecological stoichiometry is a multivariate approach that uses multiple currencies of choice—the atomic ratios of elements—as a metric [22]. Therefore, ecological stoichiometry can provide additional predictive power and complement traditional approaches [22,23]. Ecological stoichiometry considers that organisms build their bodies and maintain the entire metabolism by relying on thousands of chemical reactions [22,24]. All these reactions must be chemically balanced, as predicted by the law of conservation of mass. To put it simply, this rule is similar to the case of a single chemical reaction, in which the atomic composition of reactants is exactly the same as the atomic composition of products (see Reference [22], where this concept was taken further and described in reader-friendly form). Therefore, ecological stoichiometry refers to atoms of chemical elements whose scarcity in the environment prevents the production of biologically important organic molecules (e.g., nucleic acids, phospholipids, vitamins, and enzymes, i.e., essentially the biochemical machinery that builds every living organism, and which is required for their maintenance). Within this context, ecological interactions of organisms in an ecosystem are shaped by the quality of available food (Figure 2; [23,25,26]; see Reference [16] for a review).

Because adult bodies are already fully formed, their functionality is mainly limited by energy; however, according to the homeostatic “elemental recipe” (i.e., elemental homeostasis; [23,27]), the ability of an organism to build a fully functional adult body is influenced by the availability of body-building atoms during the juvenile stage (the changes in the chemical composition of the body during subsequent adult life are a different story). The larval diet must be nutritionally balanced in terms of the proportions of nutrients in the food being consumed; it is impossible to obtain a balanced diet by simply eating a large quantity of nutritionally imbalanced food [23,26,28]. Within this context, stoichiometric mismatch may occur between the elemental composition of the body of the consumer and its food [23,28,29]. The observed “toxic” effects (e.g., negative influences on egg production, hatching success, and development) of a particular diet on an organism may be caused by a stoichiometric mismatch, rather than by toxic substances [30]. In this way, the demand for resources is reflected in organismal stoichiometry [25].

Organisms face limitations imposed by the scarcity of organic substances, including specific amino acids, in food [31]. However, it is practically impossible for a single study to encompass the abundance of organic components that make food nutritionally balanced because of the wide diversity of such components. Therefore, previous studies focused on either (1) a specific group of organic substances (e.g., phospholipid fatty acids (PLFAs) or amino acids) or (2) the total concentrations of carbohydrates, proteins, and lipids (reviews in References [10,14,31]). Approach (1) provides valuable but limited information on the nutritional ecology of bees, and method (2) overlooks the possibility that the scarcity of specific substances in food may affect bees even if the food contains a large amount of proteins, sugars, or lipids. An alternative and complementary approach is to use the framework of ecological stoichiometry to study the constraints that unbalanced food imposes on organismal growth and development [23,25,26]. During larval development, organisms assimilate all the building blocks needed to compose the adult body. The body is built of atoms in a taxonomically specific proportion known as the organismal stoichiometry [25]. The demand for resources gathered during larval growth is reflected in the organismal stoichiometry of the adult body, and stoichiometric mismatch may occur between the atomic composition of the body and the larval food [23]. Poor nutritional balance in the pollen consumed may limit the growth and development of individuals, thus negatively influencing their populations [23,31].

## 2. Adult Bee Individuals Need Food Quantity, but Bee Populations Need Food Quality to Thrive and Prosper

A number of studies investigated the nutritional needs of wild bees considering the food quantity (e.g., Reference [32]; for a review, see References [9,10]). Within this framework, it was shown that the quantity of available food may shape the reproduction patterns and densities of wild bees (e.g., References [32,33,34]). Much less is known about the nutritional quality of pollen for wild bees. In contrast, our knowledge on the nutritional ecology of *A. mellifera* is more advanced; however, even for this species, the nutritional needs were mainly studied considering the food quantity and neglecting its quality [14,17]. This knowledge gap is reflected in conservation strategies undertaken to stop pollinator decline. As an illustration of the risk of ignoring the quality of larval food for bees, one could consider mass-flowering crops being visited by adult pollinators for high-energy food that is unbalanced for larvae. Mass-flowering crops, even if rich in nectar and pollen, serve as a monotonous diet and act as a stressor on bee health, limiting growth and development [4,16]. It was shown that mass-flowering crops dilute pollinator abundance [35]. Nonetheless, mass-flowering crops were proposed as conservation resources for wild bees, based on a collection of adult specimens from a sunflower field [36], while ignoring the fact that sunflowers produce pollen that is exceptionally low in phosphorus, thus limiting the larval growth and development of both wild bees and *A. mellifera* honey bees [16,17] (such a limitation results in toxic effects [30]). Therefore, a simplistic generalization of the nutritional needs of bee communities and populations based on the foraging of adult insects is misleading, and it may lead to devastating effects if applied in conservation strategies. For that reason, assessing and comparing the nutritional quality of food for bees is needed to understand the nutritional ecology of wild bees, and to implement more effective conservation practices.

## 3. The Application of Ecological Stoichiometry to Gain Knowledge on the Nutritional Ecology of Wild Bees

### 3.1. Ecological Stoichiometry Reflects the Economy of Nature

Mismatches between the biochemical makeup of an organism and its food influence the organism’s development, condition, health, size, longevity, and survival. The reason for this is economic and involves two components [25,26]: (1) the biochemical makeup of an organism originates in organismal traits and adaptations, and, at the same time, (2) the biochemical makeup reflects a demand for resources that must be acquired during growth to build a body equipped with these traits and adaptations. All living organisms are composed of identical building blocks, producing a great diversity of structures with diverse functions. These building blocks consist of approximately 25 chemical elements that are combined and maintained with the use of energy [23,28,37]. Nevertheless, the tissues and bodies of organisms are built from atoms in various proportions. This fact is crucial for ecological interactions, and it may shape the functioning of whole ecosystems (Figure 2; [23,28,38,39,40,41,42]). The most fundamental feature of the elements, enabling their use in ecological stoichiometry, is that specific atoms cannot be transformed into different atoms through processing by the organism. This feature distinguishes atoms from organic compounds. A consequence of this feature is that, in using atomic ratios of elements as a currency of choice, one can assume that every developing organism has, at its disposal, only the building material that its environment provides. In the case of bees, other pollen eaters, and herbivores in general, this material consists of a few elements available in excess (e.g., C, H, and O) and a deficit of others (e.g., P, N, and Na [23,43]). As a result, the mismatch between the elemental composition of pollen and the requirements of a bee imposes a limitation on the growth and development of bees, even if pollen is available in excess (Figure 1; [23,38,44,45]). Furthermore, growth and development are co-limited by the scarcity of several elements in addition to the most limiting element [37,46]. Within this context, ecological stoichiometry provides a common currency linking the ecology of organisms with life-history tradeoffs and evolutionary processes entrenched in the biogeochemical economy of life [25]. This currency is a ratio of atoms composing the bodies of organisms and their foods [23]. Ecological stoichiometry is not a new name for the physiology of micronutrients, and it does not concern the ions for which already grown organisms have physiological needs. Instead, the framework of ecological stoichiometry acknowledges the law of conservation of mass, which predicts that all the elements composing living things cannot be created from nothing, and they are available only in the amounts and ratios present in the environmentally available food from which growing organisms must build their adult bodies. Within this context, limitations experienced during the juvenile stage may influence life-history traits (e.g., adult size, adult condition, adult fertility, longevity of larval development, and larval consumption rate), and thus, an organism’s fitness. 

### 3.2. Changes in the Floral Composition of Bee Habitats Impact the Nutritional Balance of Bee Larvae Diets, Thereby Shaping Bee Populations

The quality of available resources is variable over the landscape and across time. Simultaneously, the expression and evolution of life-history tradeoffs are linked to the nutritional limitations experienced by the organism [25,47,48,49]. The nutritional needs of organisms for body-building atoms are reflected in specific elemental phenotypes of various organisms [50], and fitness-related traits necessitate tradeoffs in the presence of suboptimal food with mismatched atomic composition [25,48,51]. Resources acquired during the larval growth of a holometabolous insect are used to build its adult body. For building the body of a pupating individual with maximal fitness, there are optimal proportions of nutrients that may be allocated to specific structures in the adult body and to the cocoon. It was shown that the taxonomic variation in pollen stoichiometry limits the capability of bees to optimize diets for their progeny, thereby limiting the growth and development of the progeny [16,17]. In other words, various plant species produce stoichiometrically distinct pollen. Moreover, research showed that nutritionally diverse pollens from various plants act synergistically to influence host nutrition [52], and the loss of pollen host plants was shown to be the key factor driving wild-bee decline in the Netherlands [53]. Within this context, bee communities and populations may be shaped by the availability of key host plants that produce pollen providing a nutritionally balanced larval diet. Therefore, owing to differences between nutritional supply and demand, bee populations and communities may be influenced not by floral diversity per se, but by the floral composition of inhabited areas. Indeed, the attractiveness of wildflower mixtures for wild bees was shown to depend on several key plant species [54]. Consequently, the flourishing of a local bee population may depend on the flora community structure (for additional information, see References [55,56], which show how resource availability shapes the utilization of semi-natural habitats by insect pollinators). The stoichiometric variation in the available pollen species may affect the feeding strategy, reproduction patterns, and mortality risk of bees. However, bees may optimize the composition of collected pollen species to overcome stoichiometric mismatches by maximizing the quantities of the most limiting elements, and by minimizing the costs of food collection. Even so, the simple availability of specific plant species that produce stoichiometrically balanced pollen influences the condition and fitness of individual bees, consequently shaping their populations. If the floral composition of the bee habitat contains easily accessible but stoichiometrically unbalanced pollen, bee diversity and abundance will be negatively impacted, even if large amounts of pollen and nectar are available.

Decreasing plant diversity is thought to be one of the causes of the dwindling number of pollinators worldwide. Studies showed that various plant species interdependently shape the nutrition of *A. mellifera* honey bees [17,52] and wild mason bees [16,21], and that the foraging strategies of bumblebees may be influenced by pollen macronutrient ratios [57,58]. Similarly, the first study considering the nutritional composition of pollen collected by four species of stingless bees in southeast Asia shows preferences toward four plant taxa, even though a total of 16 taxa were recognized in the collected pollen, and several stingless bee species (Meliponini) inhabiting various parts of the world show preferences toward specific species of pollen [15]. However, even though data on pollen species collected by various bees are available and the chemical composition of these pollens is known, the majority of studies do not discuss the obtained data considering the needs of bees for nutritionally balanced diets, but focus rather on human needs and human diet supplementation [15]. Therefore, we do not know the extent to which wild-bee populations are limited by the availability of various pollen species, or how specific changes in floral composition and diversity impact the nutritional balance of bee diets. Combining knowledge of the nutritional needs of bees with data on the chemical compositions of various pollen species would allow us to identify key plant species that can help bees compose balanced diets. Studies that address the nutritional ecology of wild bees while also considering the various species inhabiting different ecosystems are crucial for understanding how nutritional limitations impact bee populations. An improved understanding of the relationship among the floral composition of a bee habitat, the nutritional quality of the available food resources, and bee population dynamics is essential for the success and sustainability of conservation efforts aimed at mitigating bee decline. 

### 3.3. Stoichiometric Niche

Different bee species prefer different pollen species (e.g., References [18,19,20]). These preferences may be stoichiometrically determined, as predicted by the concept of the “*multidimensional stoichiometric niche*” [59], based on the following: (1) since different species of consumers differ in their body multi-elemental stoichiometries, they also differ in their nutritional demands for the production of these bodies; (2) similarly, the potential food supplied in the environment differs in its multi-elemental stoichiometry, and, in the case of pollen, various plant species may offer stoichiometrically different pollen types (reviews of pollen stoichiometry: References [16,17]); (3) according to González and colleagues [59], the stoichiometric niche is the region of multivariate niche space occupied by a group of individuals with similar stoichiometries, and specific species may occupy specific niches; and (4) therefore, to obtain stoichiometrically balanced food, various bee species that differ in their multi-elemental stoichiometries might prefer various pollen species, or similar pollens in different proportions. 

### 3.4. Sexual Dimorphism in Nutritional Needs May Shape Bee Populations

Our understanding of population dynamics would benefit from incorporating data on within-population variation in experienced limitations posed on fitness and tradeoffs connected to these limitations [25,48]. Population growth and dynamics may be shaped by sexual dimorphism of nutritional optima, and the capacity of each sex to fulfill a nutritional optimum based on shared resources [48].

It was suggested that organismal stoichiometry, particularly *C:P* and *N:P* atomic ratios, is sex-dependent because of differential investment in specific sexual characters [25,48]. Thus, *C:P* ratios may be high in eggs (because of high lipid investment), enriching females in C and increasing female nutritional demand for C. Additionally, adult female bodies may be richer in P than male bodies because of substantial RNA investment. Goos and colleagues [60] showed that sexual dimorphism in stoichiometry is more sophisticated than previously predicted [25,48], and is multi-elemental, extending beyond the *C:N:P* stoichiometry.

In holometabolous insects, females rely on their larval diet as a source of matter for adequate nutritional quality for investment in future breeding [49]. Within this context, sexual dimorphism was shown in the behavioral and physiological regulation of nutrient acquisition, including preferred protein–carbohydrate intake, body nutrient growth, the rate of food intake, and the efficiency of utilizing ingested nutrients for body growth [61]. Nevertheless, our knowledge on sexual differences in behavioral, and physiological processes that regulate nutrient acquisition in capital breeding insects is limited.

Variability in food nutrient content is linked to the expression of physiological tradeoffs among multiple fitness-enhancing traits, frequently discussed at a very general level as growth, survival, and reproduction [47,49]. Available nutrients are taken in by organisms and are allocated to specific activities (e.g., foraging, growth, reproduction, maintenance, and storage), and this allocation results in the suite of traits characterizing specific organisms in a given environment [49]. However, the allocation may be a more sophisticated process. The simple distribution of nutrients available in the environment into somatic growth and building an optimal adult body, ensuring maximized fitness, is limited by both the quantity and quality of available resources [62]. This limitation is likely sex-dependent [48]. Optimal allocation of acquired nutrients does not mean the direct distribution of energy or matter to activities such as maintenance, growth, storage, and reproduction. Rather, it means that given proportions of nutrients composing acquired matter are allocated to specific traits (see examples in References [49,51,60,61]). Therefore, individuals of different sexes, relying on a nutritional supply of similar stoichiometric quality, are faced with different nutritional limitations. Consequently, to maximize their fitness, they should allocate stoichiometrically different resources to specific activities. 

Intraspecific variability in elemental stoichiometry is not well documented, and most stoichiometrically explicit models treat populations as a pool of elements, ignoring the structures of those populations. In such a way, the fact that variability among individuals can influence the outcome of resource limitation was underestimated [63,64]. The stoichiometric constraints posed on a single life-history trait of a certain life stage or sex may limit population growth [48,64] (see also References [25,63] for context), and conflict between the sexes in their ability to reach their own sex-specific optima may have important consequences for population growth [48]. For that reason, sexual dimorphism in nutritional needs should be considered in future studies on the nutritional ecology of wild bees, and ecological stoichiometry provides a ready-to-use framework for such studies (e.g., References [17,48,50,60]).

### 3.5. Solitary Bee—A Model Organism for Ecological Stoichiometry Studies

The unique biology of solitary bees enables the exact food eaten and 100% of the feces excreted during development to be collected precisely for a single specimen under natural conditions, and it allows easy preparation of laboratory feeding experiments. This capability makes solitary bees a perfect model organism for studies on ecological stoichiometry. The framework of ecological stoichiometry may be applied to any organism and to any of 20,000 bee species, regardless of their sociality, life-history traits, and foraging and breeding strategy. Solitary bees may be used as model systems for practical reasons: their nutritional ecology is simple compared with social bees, and they provide an opportunity for accurate but simple collection of all the food eaten and all the feces excreted during larval development. However, ecological stoichiometry may and should also be used to study ecological interactions of social bees with their host plants, as well as to study how nutrition may influence the ratio of different castes in a colony, as was already done in case of *A. mellifera* [17]. As an example, I now briefly describe the breeding biology of one solitary bee species native to Europe, West Asia, and North Africa, *Osmia bicornis* L. This example may be applied to any bee species inhabiting any part of the world. In the case of *O. bicornis*, the female lays its eggs in cracks or holes, such as in fractured wood and rocks or cracks in building walls, preferring empty *Phragmites* stems [65,66,67]. The nests are fine-lined, and they consist of a few-to-several dozen compartments (larval cells) that are closed on both sides. One egg is laid in each cell after a defined amount of pollen is deposited, and the female determines the sex of the egg by either fertilizing it or not [65,66]. The pollen load that the mother provides for her progeny is larger for female than for male offspring, and the cells prepared for females are provisioned first, and are, thus, located in the rear of the nest, which allows easy sex identification at any developmental stage. The pollen load is subsequently utilized by the larva during growth and development [65,68]. Larvae pupate in the summer, and bees overwinter in their cocoons as adults, and emerge the following spring [65]. All the excreta produced during the larval period are stored in a cell together with the cocoon and can be easily collected. Therefore, the whole elemental budget can be easily studied for a single specimen, from the pollen eaten by a single larva to an adult specimen that built itself and its cocoon based on this pollen, including all the excreta produced during the entire larval period. Additionally, feeding experiments performed in the framework of ecological stoichiometry would be, for practical reasons, easier to do using solitary bees as model systems than by using social bees.

## 4. Conclusions and Avenues for Future Research

Food resources are crucial for animal growth, development, and population dynamics [10], and the reduced diversity of plants producing bee food was suggested as a factor responsible for the pollinator crisis [3,9,69,70]. Considering this, taxonomical variance in pollen stoichiometry may limit pollen-eater development, directly linking the loss of floral diversity to bee nutritional needs [16,17]. Therefore, considering the need to overcome nutritional mismatches that result from a stoichiometrically unbalanced diet will lead to a better understanding of the decline of pollinators, and may result in more successful intervention strategies.

The floral composition of the bee habitat, and especially the occurrence of key species that provide a nutritionally balanced larval diet, may be a factor influencing bee populations. Thus, not only the quantity, but also the quality, of food sources for bees should be considered in intervention strategies aimed at improving the nutritional base for bees. Simply planting random plant species that offer pollen or nectar in large quantities is not a good practice. However, bee-friendly plants are often defined based on the quantities of nectar and pollen that they produce, as well as on the visitation rates of adult insects that forage for energy. This conception is misleading, and incorporation of taxonomically and sexually specific nutritional requirements of wild bees into conservation strategies could improve the nutritional base for bees.

To gain an adequate understanding of ecosystems’ current and past behavior, and to better anticipate future changes, knowledge is needed regarding (1) the limitations imposed on the life histories of organisms inhabiting considered ecosystems, (2) the relationships between these organisms, and (3) the dynamics of their populations. One important factor in this respect is the flow of energy and the cycling of matter [23,28,41]. Ecological stoichiometry provides a ready-to-use framework for studying how the balance of energy and matter affects organisms and their interactions in ecosystems [23]. This framework enables an easy and accurate comparison of the nutritional demands of bee larvae for body-building nutrients with the supply of these nutrients in an inhabited environment. The stoichiometric mismatch between larval nutritional demand and supply may negatively affect bee communities and populations. Therefore, I propose the application of ecological stoichiometry in studying the nutritional ecology of wild bees.

A recent study noted that, in the scientific literature, it is easier to find a population’s genome than its elemental composition [37]. The dependency of bees on pollen stoichiometry, which is taxonomically variable, is yet to be studied, except in two works on *O. bicornis* and *A. mellifera*, and various taxa of pollen [16,17]. At the same time, the concept of a “stoichiometric niche” was introduced and defined as the region of a multivariate niche space occupied by a group of individuals where the axes represent their elemental content [59]. If a consumer is not able to find food fitting its stoichiometric niche, a limitation is imposed on its growth and development, negatively influencing its fitness, and the whole population may be negatively impacted in this way [25,26]. Since a considerable knowledge gap exists concerning the atomic composition and stoichiometric relationships between wild bees and their potential food sources, the following questions should be answered in future studies to gain knowledge on the nutritional ecology of wild bees that could improve conservation strategies:

1. How do various taxa of pollen differ in their stoichiometry?
2. How do various species, and different sexes and castes of bees differ in their stoichiometry, and therefore, how different are their nutritional demands? Which scarce elements limit the growth and development of different sexes and castes, and of various species of bees?
3. Following (1) and (2), which specific key host plant species that produce stoichiometrically desirable pollen allow bees to balance their diets?

Understanding the demand for a nutritionally balanced diet in growing bees is critical for understanding ongoing changes in wild-bee populations worldwide, and for predicting future changes connected with transformations of bee-habitat floral composition. Within this context, experimental studies manipulating the nutritional supply of various pollen species are needed to elucidate ecological interactions between bees and their known and potential host plant species. To enable us to fully understand the nutritional aspects of bee–plant interactions, these studies might use the framework of ecological stoichiometry. This would lead to an understanding of the most basic mechanism shaping the nutritional ecology of bees—balancing the larval diet to enable its growth, development, and pupation into the adult body equipped with all the structures needed for maximized fitness. At the same time, as a complementary approach, the concentrations of the chosen organic substances (e.g., important PLFAs, amino acids, vitamins, etc.) in studied pollens could be measured to detect mechanisms shaping another level of possible limitations posed on developing larvae. Finally, to fully understand the bee nutritional ecology, gut-dwelling symbionts should be considered, including their needs for a stoichiometrically balanced diet, as well as for specific organic compounds. These three complementary approaches could result in an improved understanding of the behavior of ecosystems, an improved prediction of how changes in floral structure affect pollen-eating pollinators, and improved strategies for pollinator conservation and management.
